# IHF stabilizes pathogenicity island I of uropathogenic *Escherichia coli* strain 536 by attenuating integrase I promoter activity

**DOI:** 10.1038/s41598-020-66215-2

**Published:** 2020-06-10

**Authors:** Marco Chittò, Michael Berger, Petya Berger, Luisa Klotz, Peter Dröge, Ulrich Dobrindt

**Affiliations:** 1 Institute of Hygiene, University of Münster, Münster, Germany; 2 Interdisciplinary Center for Clinical Research, University of Münster, 48149 Münster, Germany; 3 Department of Neurology with Institute of Translational Neurology, University Hospital Münster, 48149 Münster, Germany; 4 School of Biological Sciences, Nanyang Technological University, 637551 Nanyang, Singapore

**Keywords:** Microbiology, Microbial genetics, Pathogens

## Abstract

Pathogenicity islands (PAIs) represent horizontally acquired chromosomal regions and encode their cognate integrase, which mediates chromosomal integration and excision of the island. These site-specific recombination reactions have to be tightly controlled to maintain genomic stability, and their directionality depends on accessory proteins. The integration host factor (IHF) and the factor for inversion stimulation (Fis) are often involved in recombinogenic complex formation and controlling the directionality of the recombination reaction. We investigated the role of the accessory host factors IHF and Fis in controlling the stability of six PAIs in uropathogenic *Escherichia coli* strain 536. By comparing the loss of individual PAIs in the presence or absence of IHF or Fis, we showed that IHF specifically stabilized PAI I_536_ and that in particular the IHFB subunit seems to be important for this function. We employed complex genetic studies to address the role of IHF in PAI I_536_-encoded integrase (IntI) expression. Based on different YFP-reporter constructs and electrophoretic mobility shift assays we demonstrated that IntI acts a strong repressor of its own synthesis, and that IHF binding to the *int*I promoter region reduces the probability of *int*I promoter activation. Our results extend the current knowledge of the role of IHF in controlling directionality of site specific recombination reactions and thus PAI stability.

## Introduction

Urinary tract infection is the most frequent type of nosocomial, but also community-acquired bacterial infection and in general the most frequent type of bacterial infections in women^1^. The vast majority of these infections is caused by uropathogenic *Escherichia coli* (UPEC) and are therefore from the perspective of general health, but also due to economic reasons, the most important problem associated with this bacterial species^2–4^. *E. coli* factors that facilitate the colonization of the urinary tract, e.g. fimbrial adhesins as well as toxins are often encoded on large mobile genomic regions designated pathogenicity island (PAIs). Seven archetypal PAIs have been described in uropathogenic model strain *E. coli* 536 and for six of these PAIs experimental data is available showing that they differ in their genetic stability^5–8^. These PAIs carry virulence and fitness-associated genes that contribute to the bacterial pathogenesis and/or provide a survival advantage in the host^5,9,10^. PAIs form a distinct class of integrative genetic elements^11–13^, which exhibit several defining features, including the presence of a P4-type integrase of the tyrosine recombinase family^14–16^. A comprehensive understanding of the mechanisms that result in the mobilization of PAIs is necessary for the development of strategies to prevent the generation of new bacterial strains with unknown pathogenic potential and for approaches that aim at the attenuation of bacterial virulence.

The chromosomal integration and excision of PAI I_536_-VI_536_ of *E. coli* strain 536 was shown to be dependent on PAI encoded P4-like integrases^17^. P4-like integrases belong to the family of tyrosine recombinases that are characterized by the presence of a conserved C-terminal Tyr residue^18^. The integrase of the bacteriophage P4 operates by integrating the phage DNA into the host chromosome via site-specific recombination^19,20^, which requires the P4 integrase, the phage attachment site *attP*, and the chromosomal attachment site *attB*^19,21^. The site-specific recombination reaction between the *attP* and *attB* sites involves DNA cleavage, formation of a Holliday junction, and rejoining of DNA fragments^22–24^. In the case of bacteriophage P4, the integration site resides within a leucyl-tRNA locus^21,25^. After integration of the bacteriophage genome into the bacterial chromosome, the P4 *int* gene is located in the proximity of the *attL* site^23^. In addition to the integrase itself, bacteriophages such as ʎ, P2 and P4 require accessory host proteins, such as the integration host factor (IHF) and the factor for inversion stimulation (Fis) for integrative recombination. These accessory DNA bending proteins bridge between distinct DNA sites, thereby implementing integrative or excisive recombination^26–28^.

IHF is a small hetero- and homo dimer forming DNA binding protein composed of two subunits IHFA and IHFB^29,30^. Upon binding, IHF is known to bend DNA^29,31^. It is generally involved in a variety of DNA-depended processes including site-specific DNA recombination, transposition, transcriptional regulation and DNA replication^32–34^. The bacteriophage-encoded integrase and IHF are known to form a complex during bacteriophage genome integration, the so-called intasome^32,35^. After integration, expression of the integrase gene must be tightly regulated, as the integrase is also catalyzing the excision reaction^25^. For the bacteriophage P4, it was shown that the integrase is functioning as a repressor of its own promoter^25^. In addition, the P4-encoded excisionase (Vis) was shown to be involved in the repression of the integrase promoter (P*int*P4)^27^. The P*int*P4 contains also an IHF binding site, but if IHF plays a role in the regulation of *int*P4 expression is not clear^27^. In addition to IHF, the host protein Fis appears to be involved in the integrative process of temperate bacteriophages^36^. We therefore decided to test if these proteins are involved in the excision process of PAI I_536_-VI_536_ and constructed isogenic *ihfA/B* and *fis* mutants of *E. coli* 536 reporter strains for the measurement of PAI I_536_-VI_536_ stability^37^. We found that Fis only plays a minor stabilizing role on individual PAIs. Likewise, IHF played a minor role in stabilizing PAI II_536_-VI_536_. However, the stability of PAI I_536_ was severely compromised in cells lacking IHF.

## Results

### Role of Fis and IHF in chromosomal stability of PAI I_536_-VI_536_ of *E. coli* strain 536

**Figure 1:**
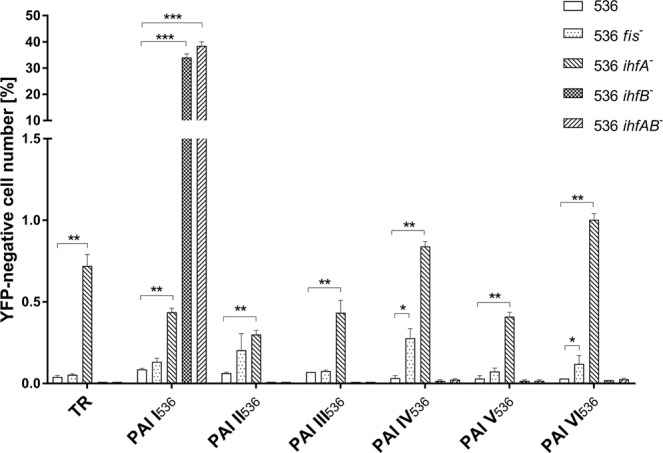
Impact of Fis and IHF on the stability of six archetypal PAIs of *E. coli* strain 536. Fraction (%) of YFP-negative cells in cultures of *E. coli* strain 536 and its 536 *fis*^*−*^, *ihfA*^−^, *ihfB*^−^ and *ihfAB*^*−*^ mutants. Each of the columns represents the average of three biological replicates, in which 10^7^ cells per sample were analyzed (*P < 0.05; **P < 0.01; ***P < 0.001).

In order to address the question whether Fis and/or IHF play a role in stabilizing PAI I_536_-VI_536_ of *E. coli* strain 536, we constructed isogenic *fis* and *ihfA/B* mutants in our previously described reporter strains that allow for the measurement of the stability of PAI I_536_-VI_536_^37^. Briefly, we inserted a P*dps-yfp* module in intergenic regions of the six PAIs of *E. coli* strain 536. This module allows expression of YFP (yellow fluorescent protein) when the cells enter stationary phase and is inactive during logarithmic growth. In addition, we inserted the module in the conserved chromosomal control position TR. As we have shown previously, the control position TR was functionally equivalent to five other tested chromosomal positions throughout the *E. coli* K-12 chromosome with respect to the expression of the P*dps* module^38^. Here, we used the *yfp*-based module in order to distinguish cells that carry the module from those that have lost it simply by analyzing the YFP signal by flow cytometry. We found that whereas Fis might play a minor, but significant role in the stabilization of PAI IV_536_ and PAI VI_536_ under standard laboratory growth conditions (Fig. 1), IHFA/B did not negatively affect the stability of PAIs of *E. coli* strain 536, except for PAI I_536_ (Fig. 1). We found that almost 40% of the bacteria had lost PAI I_536_ in the *ihfA/B* mutant as judged by the absence of YFP signal in a large part of the population. To validate that the YFP-negative cells had lost PAI I_536_, we plated aliquots of an overnight (ON) culture on LB plates, incubated overnight and examined the obtained colonies by fluorescence microscopy. We subsequently analyzed the colonies by PCR and Sanger sequencing. In addition, we sorted 100.000 YFP-negative cells and used their genomic DNA as PCR template. Sequencing of the PCR product revealed exactly the same “scar sequence” resulting from chromosomal PAI I_536_ deletion as the sequencing of the PCR product of individual YFP-negative colonies, which indicated that the PAI excision process was very precise (Fig. S1). As PAI I_536_ is also encoding the toxin α-haemolysin, one important virulence factors of *E. coli* strain 536, we decided to analyze the IHF-dependent regulation of the stability of PAI I_536_ in more detail.

### Lack of *ihfB* is sufficient to destabilize PAI I_536_

**Figure 2:**
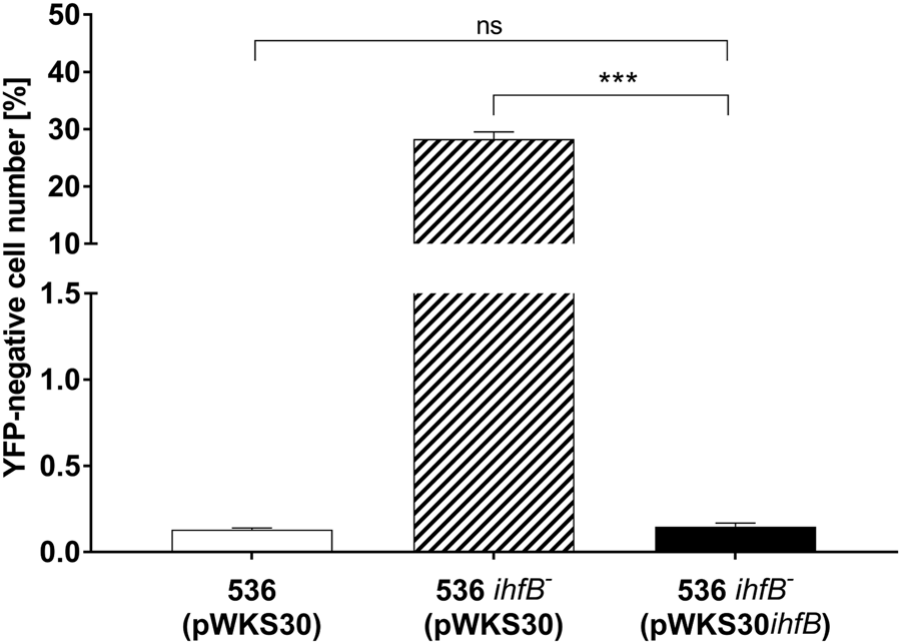
The deletion of *ihfB* is sufficient to destabilize PAI I_536_. Fraction (%) of YFP-negative cells in cultures of wild type *E. coli* strains 536 and 536 *ihfB*^*−*^ containing the vector control and the *ihfB* complemented *E. coli* 536 *ihfB*^*−*^. Each of the columns represents the average of three biological replicates in which 10^7^ cells per sample were analyzed (*P < 0.05; **P < 0.01; ***P < 0.001).

Next, we analyzed the stability of PAI I_536_-VI_536_ in cells lacking either functional *ihfA* or *ihfB*. We found that loss of functional *ihfA* increased the number of YFP-negative cells for all reporter strains, including the chromosomal control position TR (Fig. 1)^37,38^. In contrast, the absence of *ihfB* was specifically destabilizing PAI I_536_. Notably, the number of YFP-negative cells in the *ihfB* mutant was in the same range as observed with the *ihfA/B* double knockout mutant, suggesting that IHFB could play a greater role than IHFA in IHFAB-dependent stabilization of PAI I_536_ (Fig. 1). Importantly, complementation with *ihfB* restored the stability of PAI I_536_ (Fig. 2).

### An IHF binding site in the promoter region of *int*I is required for the stabilization of PAI I_536_

**Figure 3:**
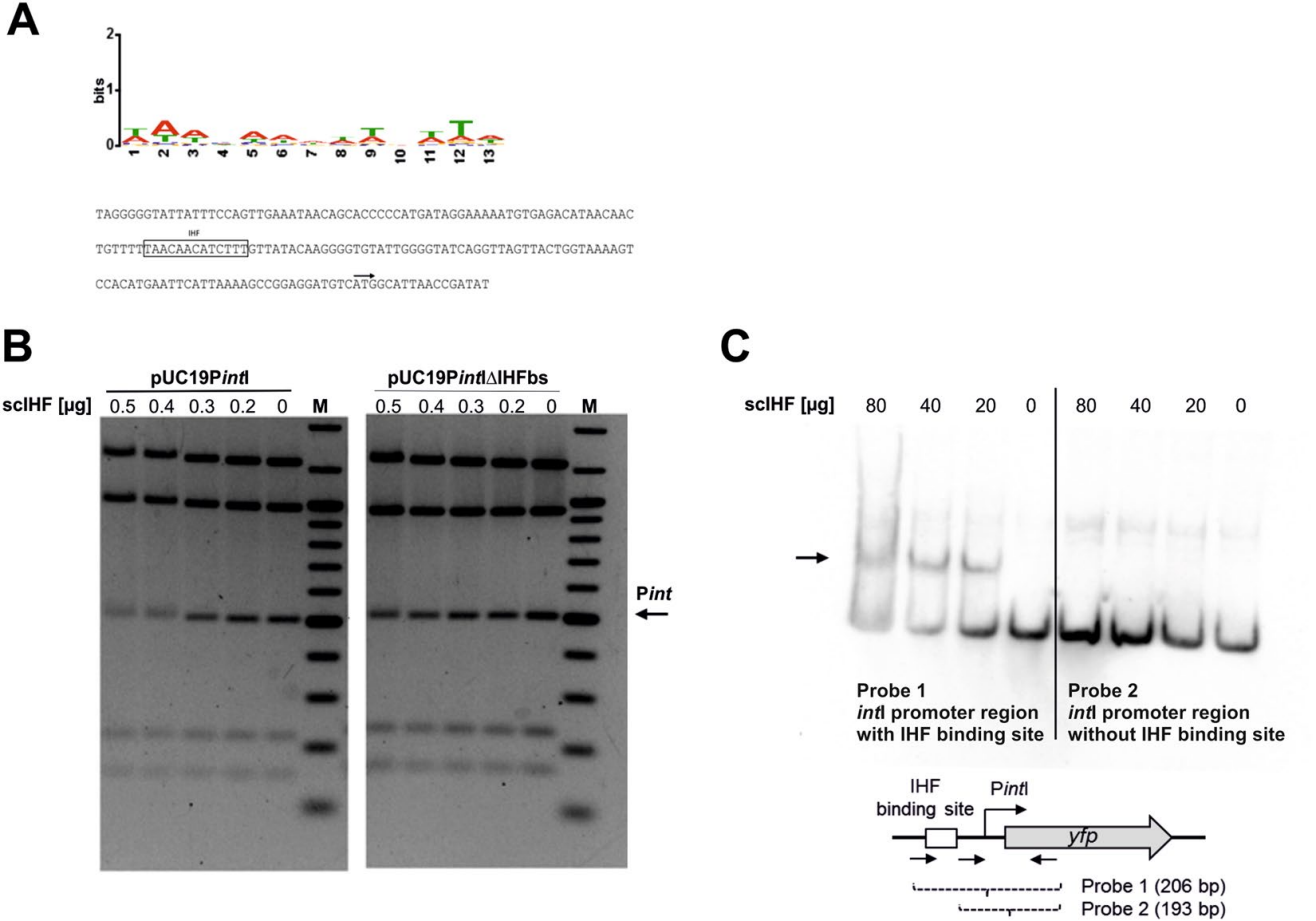
IHF binds to a high affinity binding site in the *int*I promoter region. (**A**) Sequence logo used to computationally predict IHF binding sites in the vicinity of *int*I-VI (up) and nucleotide sequence upstream of the *int*I gene (down). The binding site sequence logo was created using MEME^39^ and the experimentally identified IHF binding sites listed in RegulonDB^38^. Annotated are the main features of the *int*I gene: predicted IHF binding site (box), start codon of the coding sequence of *int*I (black arrow). **(B)** Analysis of scIHF binding to the *int*I upstream region by electrophoretic mobility shift assay (EMSA). Single-chain IHF bound specifically to the promoter fragment (P*int*I) only in the presence of the IHF binding site (left), but not when the binding site was absent (right). M, DNA size marker. For this figure, the original gel has been cropped and the arrangement of the two parts of the gel representing either pUC19P*int*I or pUC19P*int*I ΔIHFbs has been reversed. The original agarose gel is provided in Supplementary Figure S6. **(C)** Digoxygenin-labeled probes comprising the *int*I upstream region with or without IHF binding site (25 ng) were incubated with or without purified scIHF (80 µg, 40 µg, 20 µg; 0 µg). Single-chain IHF bound specifically to the promoter fragment (P*int*I) only in the presence of the IHF binding site (left), but not when the binding site was absent (right).

An IHF binding site in the promoter region of *int*P4 with unknown function has previously been described^27,39^. We therefore performed a computational analysis in order to screen for potential high affinity binding sites for IHF in the upstream region of the coding sequences of integrase I of *E. coli* strain 536 (Fig. 3A).

In order to test, if IHF is binding to the predicted site in the promoter region of *int*I, we overexpressed and purified scIHF, a genetically engineered and well-characterized single-chain IHF variant that functions like the wild-type heterodimer *in vitro* and *in vivo*^29,40,41^ (Fig. S2). We cloned the upstream region of *int*I with or without the potential IHF binding site into the plasmid pUC19, digested the plasmid and tested for *in vitro* binding of scIHF using electrophoretic mobility shift assays. As shown in Fig. 3B, the mobility of restriction fragments of the plasmid including the *int*I promoter (P*int*I) fragment lacking the IHF binding site, was not affected at the scIHF concentrations tested. In contrast, the wild type *int*I promoter fragment showed a scIHF concentration-dependent diffuse shift indicating a specific and stable scIHF-*int*I promoter interaction. Additionally, we investigated the interaction of scIHF with shorter DNA probes comprising the *int*I upstream region with the predicted IHF binding site. In one of the probes this binding site was destroyed. These 3′ digoxygenin-labeled probes were incubated with different amounts of purified scIHF, and a clear shift of the DNA band was only observed with the probe carrying the IHF binding site. In case of the probe with the mutated IHF binding site, no DNA shift was visible (Fig. 3C).

### The IHF binding site is affecting the stability of PAI I_536_*intrans*

**Figure 4:**
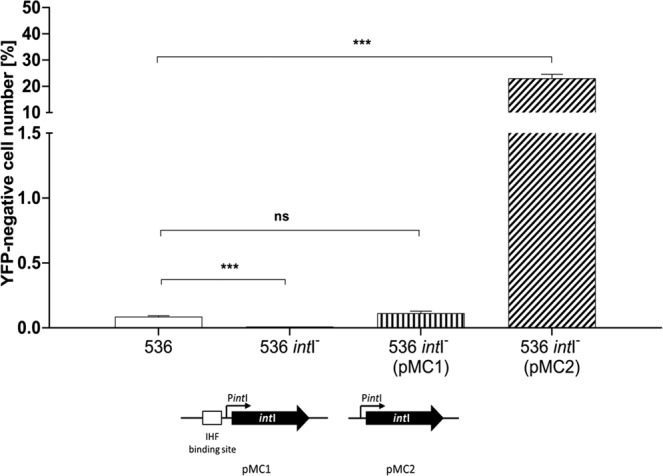
The IHF binding site is operational *in trans*. Fraction of YFP-negative cells in cultures of *E. coli* strain 536, its isogenic *int*I^−^ mutant as well as in strain 536 *int*I^−^ complemented with a plasmid-encoded copy of *int*I containing the IHF binding site (pMC1) or complemented with a plasmid-encoded copy of *int*I lacking the IHF binding site (pMC2) in percent. Each of the columns represents the average of three biological replicates in which 10^7^ cells per sample were analyzed. (*P < 0.05; **P < 0.01; ***P < 0.001; schematic representation of the main features of pMC1 and pMC2 are shown below the graph).

The presence of the IHF binding site in the upstream region of *int*I suggested that IHF might stabilize PAI I_536_ by regulating *int*I expression. However, it was also possible that IHF as part of the intasome was stabilizing PAI I_536_ by inhibiting the excision process itself. If the latter would be the case, the presence or absence of the IHF binding site should not have affected PAI I_536_ stability, when *int*I is expressed *in trans*. We therefore cloned *int*I with IHF binding site (pMC1) and without the IHF binding site (pMC2) into a low copy vector and measured the stability of PAI I_536_ in *E. coli* strain 536 *int*I^−^. As shown in Fig. 4, the deletion of *int*I stabilized the island as judged by the almost undetectable YFP-negative cells when compared to the wild type as described previously^17,37^. Complementation of strain 536 *int*I^*−*^ with pMC1 decreased PAI I_536_ stability back to wild type levels. However, complementation of *E. coli* 536 *int*I^*−*^ with pMC2 decreased the stability of PAI I_536_ in the same order of magnitude as the *ihfB* mutation. This indicated that the major function of IHF in this system was indeed the regulation of *int*I expression (compare Fig. 4 to Fig. 1).

### Integrase I is repressing its own promoter

**Figure 5:**
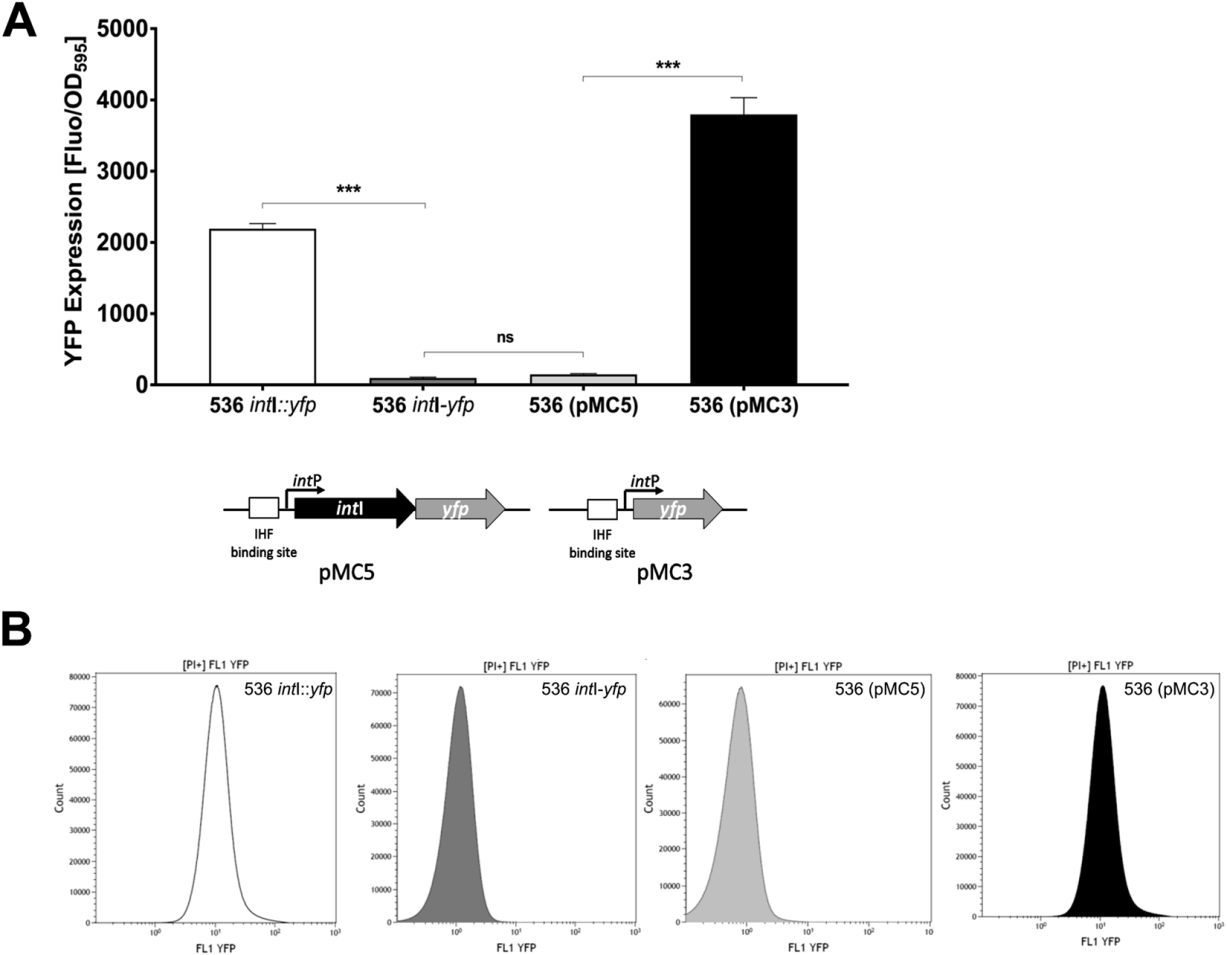
Integrase I of *E. coli* 536 is subject to autoregulation and represses its own promoter. (**A**) YFP fluorescence signal of *E. coli* strains 536 *int*I::*yfp* (chromosomal promoter fusion), 536 *int*I*-yfp* (chromosomal operon), and *E. coli* strain 536 (pMC5) that encodes an artificial plasmid-encoded *int*I*-yfp* operon, and *E. coli* strain 536 (pMC3) with a plasmid-encoded *int*I::*yfp* fusion normalized to the cell density (OD_595_). The columns represent the average of three biological replicates (*P < 0.05; **P < 0.01; ***P < 0.001). **(B)** YFP fluorescence signal distribution within the corresponding bacterial populations (10^7^ cells per sample were analyzed).

We therefore decided to analyze a chromosomal *int*I promoter-*yfp* transcriptional fusion (536 *int*I*::yfp*) in more detail^37^. Fluorescence measurements revealed an overall relatively high level of YFP expression (Fig. 5A) and a flow cytometric analysis of the bacterial population showed that this signal was overall normally distributed within the population (Fig. 5B). Despite the fact that the bacterial population also contained a very small fraction of highly fluorescent cells, the chromosomal 536 *int*I*::yfp* fusion was unique with respect to the other chromosomal integrase promoter-*yfp* fusion constructs (P*int*II to P*int*VI*-yfp*) of *E. coli* strain 536, as none of these showed such a basal, but readily detectable fluorescence signal^37^. As all chromosomal *int* promoter-*yfp* fusions were constructed in an identical way, i.e. by precisely replacing the open reading frame of the integrase genes by the reporter gene *yfp*, this suggested that the regulation of *int*I expression was distinct from that of *int*II to *int*VI. However, if the stability of PAI I_536_ was, at least in part, regulated at the level of *int*I expression, it was not clear how an overall normally distributed integrase I expression could result in the observed rare event of PAI I_536_ excision. We therefore speculated that an important factor for the regulation of *int*I expression was still missing in our reporter system for *int*I expression. As the integrase of bacteriophage P4 was shown to repress its own transcription, we decided to test the function of IntI in *int*I regulation by re-introducing *int*I into the system^27^. In order to do so, we first constructed an artificial operon with the reporter gene *yfp* transcriptionally fused downstream of the coding region of *int*I (536 *int*I-*yfp*). Afterwards we cloned the artificial operon into a low copy vector (pMC5).

The comparison of the integrase promoter activity of the artificial operon in its native chromosomal position with pMC5 did not show any significant differences, both at population average as well as on overall fluorescence signal distribution within the population (Fig. 5A,B). However, when compared to the direct 536 *int*I::*yfp* fusion, the artificial operon showed a significant reduction of the population average fluorescence signal indicating that expression of the integrase I protein is necessary for the repression of its own synthesis (Fig. 5A). The comparison of the population average fluorescence signal when *int*I*::yfp* was localized on a low copy vector (pMC3; Fig. 5A, black bar) with pMC5 (Fig. 5A, light grey bar) showed as well a significant and even more pronounced reduction for the artificial operon. As for the chromosomal 536 *int*I*::yfp* fusion, flow cytometric measurements showed that the relatively strong fluorescence signal produced by pMC3 was normally distributed (Fig. 5B). In contrast the vast majority of cells containing pMC5 did not show a detectable fluorescence signal (Fig. 5B light grey).

### IHFB is reducing the probability of *int*I promoter activation

**Figure 6:**
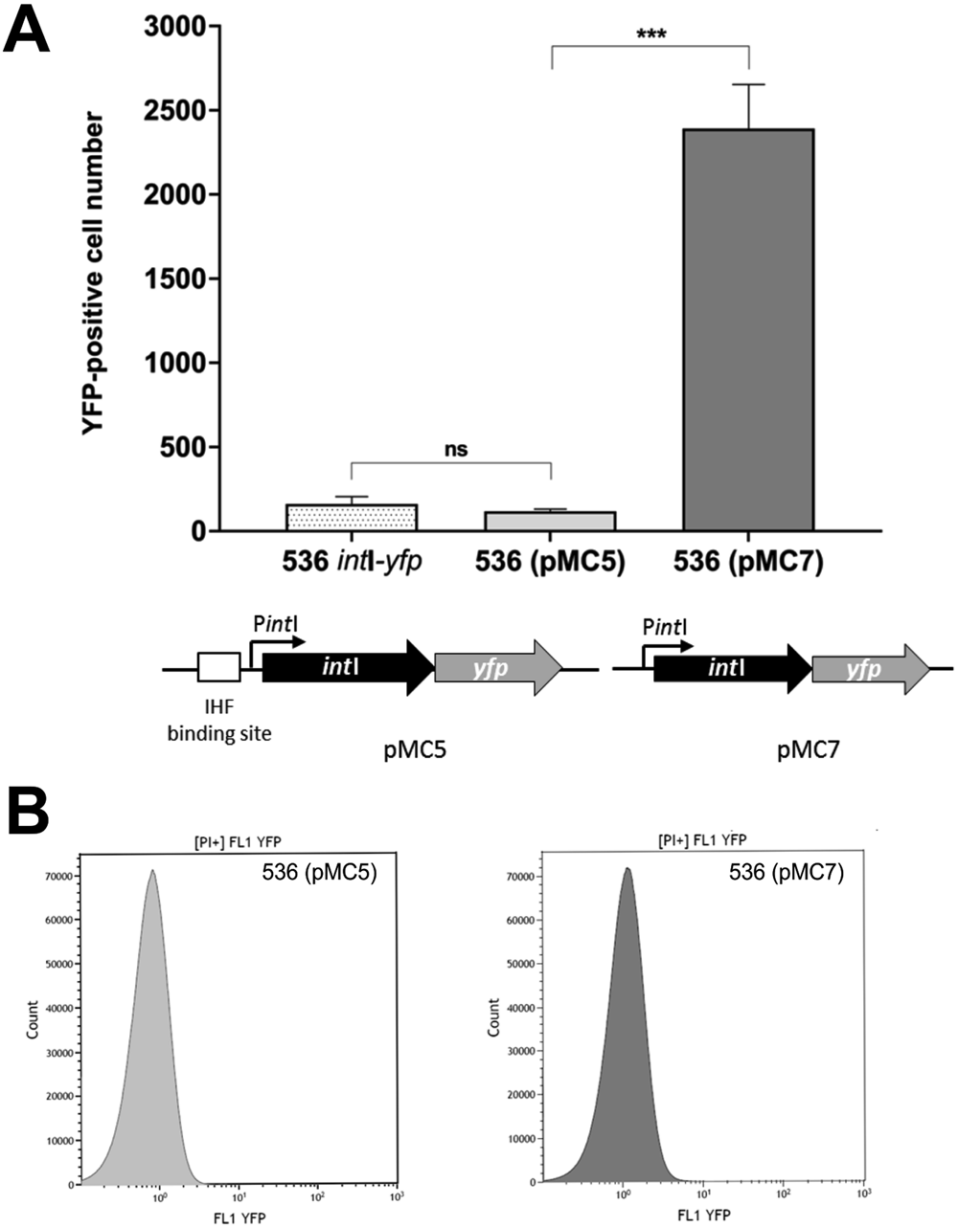
Impact of the presence of the IHF binding site on *int*I promoter activity in *E. coli* strains 536 (pMC5) and 536 (pMC7). (**A**) Number of YFP-positive cells in the populations of *E. coli* strains 536 *int*I-*yfp*, 536 (pMC5) and 536 (pMC7). The columns represent the average of three biological replicates, in which 10^7^ cells per sample were analyzed (*P < 0.05; **P < 0.01; ***P < 0.001). (**B**) Distribution of the YFP fluorescence signal in the populations of *E. coli* strains 536 (pMC5) (grey) and 536 (pMC7) (black), which lacks the IHF binding site upstream of *int*I. In both populations 10^7^ cells were analyzed per sample.

The artificial operon containing *int*I now showed characteristics that were similar to what we had observed earlier for P*int*II to P*int*VI, i.e. an almost non-detectable population average fluorescence signal and a very small subpopulation of highly fluorescent cells^37^. We therefore decided to investigate the role of the IHF binding site in the artificial operon containing *int*I and cloned the artificial operon lacking the IHF binding site into a low copy plasmid (pMC7) and analyzed the sample by flow cytometry. As shown in Fig. 6A,B, the overall number of fluorescent bacteria was low and both samples showed a similar signal distribution. However, *E. coli* strain 536 containing pMC7 that lacks the IHF binding site showed an approximately 20-fold higher number of cells with high fluorescence signal than *E. coli* 536 containing pMC5 (Fig. 6A). Notably, the same analysis of pMC5 in *E. coli* strain 536 *ihfB*^*-*^ resulted in very similar numbers of cells with high fluorescence signal indicating that one of the main functions of IHF in regulating *int*I expression is to reduce the probability of full promoter activation (compare Fig. 6A to Fig. S3B).

## Discussion

The PAIs of UPEC strain 536 are an example for horizontally acquired genetic material that has equipped the host strain with virulence and fitness traits^8,10,22,42–44^. A better understanding of the mechanisms involved in integration and stabilization of newly acquired genetic material is necessary to design strategies for the prevention or reversion of these processes. Especially in the management of milder bacterial infections, drugs that attenuate a pathogen during an infection, e. g. by stimulating the excision and loss of PAIs, might be a more sustainable approach than classical antibiosis, as such drugs would be expected to impose much less selective pressure on the bacteria. For *E. coli* strain 536 it is known that PAI I_536_-VI_536_ display different levels of stability and that the difference is, at least in part, dependent on the activity of the P4-like integrase genes^8,17,37^.

In this study we investigated in detail the effect of the two nucleoid-associated proteins Fis and IHF in the stabilization process of PAI I_536_-VI_536_, as these proteins are known to be important host factors for temperate bacteriophages. We found that Fis might play a minor role in the stabilization of individual PAIs (Fig. 1), while IHFA/B showed a strong effect specifically on the chromosomal stability of PAI I_536._ Deletion of *ihfB* was sufficient to destabilize PAI I_536_ (Fig. 1). Our observation, which suggests that IHFB, most likely in the form of a homodimer, was sufficient to stabilize PAI I_536_, is consistent with previous studies that indicated a more important *in vivo* role for IHFB homodimers than for IHFA homodimers^30,45,46^.

**Figure 7:**
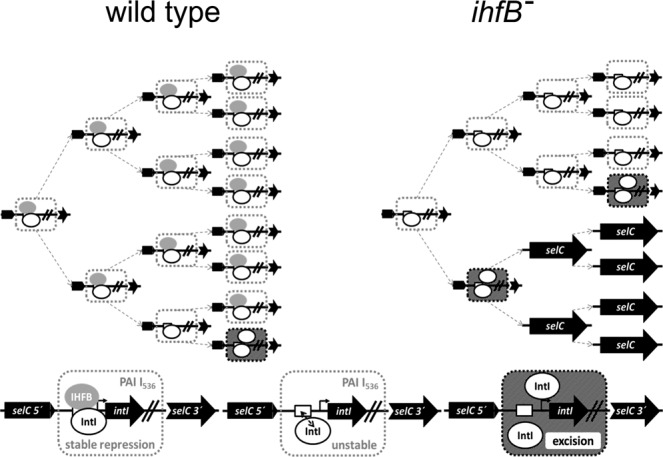
Increased frequency of *int*I promoter activation in *E. coli* strain 536 *ihfB*^*−*^ results in a rapid loss of PAI I_536_. Model for the regulation of *int*I expression by IntI and IHFB. In *E. coli* strain 536 (left) the expression of *int*I is strictly regulated by IntI and IHFB. In *E. coli* strain 536 *ihfB*^*−*^ (right) the probability for *int*I activation is higher than in the wild type. This results over time in a significantly higher fraction of cells that have lost PAI I_536_. For details see text.

We provide experimental evidence that IHF was stabilizing PAI I_536_ via regulation of *int*I expression by our observation that the IHF binding site mediated the observed phenotype *in trans* (Fig. 4). IHF can regulate transcription by different mechanisms. Usually, IHF binding modulates the DNA architecture and can thus affect the accessibility of promoter structures. Transcription can be induced when IHF binding leads to DNA bending, which allows the RNA polymerase to interact directly with a transcriptional regulator bound in a distant DNA region^47^. IHF-mediated DNA bending can also facilitate the interaction of the C-terminal domain of the RNA polymerase α subunit with upstream promoter elements, thereby inducing transcription^48^. In addition, open complex formation can be facilitated due to IHF binding^49^. If in case of PAI I_536_ the IHF binding site would have had a function exclusively in the excision process, its episomal presence or absence would not have affected the stability of PAI I_536_ to the same extent as the *ihfB* deletion. We showed that the IntI protein is repressing its own expression (Fig. 5). The regulation of *int*I by IntI in conjunction with IHF can therefore explain the observed increased frequency of the loss of PAI I_536_ in cells lacking IHF (Fig. 7): In wild type cells, IntI is repressing its own synthesis and a functional collaboration with IHF is further reducing the probability of the activation of the *int*I promoter. In a small subpopulation of cells, IntI may not form a stable complex with IHF on the promoter, and if additional IntI is produced, PAI I_536_ is excised. In the *ihfB* mutant, the repression complex involving DNA, IHFAB heterodimer and IntI cannot be formed, which results in an overall higher frequency of *int*I promoter activation, and production of additional IntI is resulting in PAI I_536_ excision. Over time, this results in the observed substantial fraction of cells lacking PAI I_536_ within the population. If our model depicted in Fig. 7 is correct, it has several interesting implications. First, if the stability of PAI I_536_ is so strictly regulated by the amount of IntI, the model would predict that there is virtually no unbound IntI available. This means that an extra copy of the *int*I promoter would be deregulated in the *E. coli* 536 wild type strain as well as in mutants lacking *int*I, which is exactly what we observe (Fig. S5). In contrast, if an extra copy of *int*I is reintroduced into the transcription unit itself, the promoter is again under strict autorepression (Fig. 5). Such a tight repression also indicates a very precisely ordered sequence of events for the activation of this promoter, which may be further elucidated by time-resolved *in vitro* footprinting techniques in the future.

Why is it so important for genome plasticity and PAI stability that integrase expression is strictly regulated? Lysogenic bacteriophages employ site-specific recombination in order to integrate into or excise from the genome of their host. The underlying mechanism has been resolved for several model phages and is considered to be identical for all temperate bacteriophages^23,25,26^. The architecture of the intasome directs integration or excision of the phage genome^24^. Accordingly, the correct stoichiometry of the different components of the recombinogenic complex, including the integrase, recombination directionality factor (RDF), and accessory factors such as IHF, is important for determining the direction of the recombination event^50^. As the *att* sites, which flank chromosomal islands or prophage genomes differ from each other in their organization and number of protein binding sites^20,51^, it can be expected that the assembly process and overall structure of the recombinogenic complex differs between individual phage families and PAIs. A tightly balanced integrase level necessary for recombination may result from structural requirements of the intasome assembly^50^. This may explain why expression of the integrases is tightly, but not uniformly regulated in all islands in UPEC strain 536. Panis and co-workers suggested that many integrase genes of prophages are negatively autoregulated by itself and by a RDF due to overlapping *attL* and integrase promoter regions. RDF binding to the *attL* site prevents binding of the RNA polymerase to the −35 region of the integrase promoter thus controlling the integrase gene expression^52^. In case of PAI I_536_, the IHF-dependent regulation of *int*I expression may represent a similar mechanism to modulate the ratio of proteins involved in intasome formation. In addition to the integrase, which may be expressed at different levels, intasome formation could also be controlled via the expression level of other proteins involved, such as IHF. The fact that we identified an IHF binding site upstream of *int*I and demonstrated that this site is specifically recognized by scIHF *in vitro* (Fig. 3) supports this hypothesis. A similar role of IHF was also described for bacteriophage HP1 in *Haemophilus influenzae* Rd, where IHF binding to the *attB* and *attP* sites as well as to the upstream region of the integrase gene promoted integrase expression and the rate of integrative recombination^53^. Also the regulatory region of the bacteriophage P4 integrase gene was shown to contain a high affinity IHF binding site^27,54^.

Could the role of IHF in PAI I_536_ instability reflect unique aspects of the regulation of PAI I_536_-associated genes? Although IHF was discovered because of its requirement for bacteriophage λ recombination, it also plays an important role as a transcription factor and is itself regulated such that its intracellular concentration increases upon entry of the stationary phase. In *E. coli* and *S. enterica* serovar Typhimurium, IHF is involved in controlling the transcription of more than 100 genes of various functions^55–57^. Comparison of the transcriptome of *ihfA*, *ihfB* and *ihfAB* mutants of *S*. Typhimurium indicated that the single and double deletion mutants had overlapping, but not identical expression profiles suggesting that IHFA and IHFB homodimers can have differential functions^56^. Mangan and colleagues reported that IHF contributes to the coordinated and synchronized expression of main virulence factors of *S*. Typhimurium including the type three secretion systems encoded on SPI-1 and SPI-2 or of flagella^56^. Horizontally acquired DNA is often subjected to “xenogenic silencing” mediated by H-NS^58,59^. In *S*. Typhimurium IHF reduces H-NS-mediated silencing when cells enter the stationary phase, thereby modulating the topology of repressed promoters and the accessibility for other regulators^60^. Whether the specific importance of IHF for *int*I expression and PAI I_536_ stability represents an aspect of a fine-tuned bacterial regulatory network accompanied with improved fitness or pathogenicity requires further analyses. PAI I_536_ encodes among other gene products important UPEC virulence factors, such as α-haemolysin, F17- and CS12-like fimbrial adhesins^6^. These virulence factors are not known to be expressed in an IHF-dependent manner. Studies on the influence of IHF on (PAI-associated) gene expression as well as on the time-resolved, growth phase-specific regulation of gene expression on individual PAIs in UPEC 536 are not yet available, which could explain the unique regulatory mechanism controlling PAI I_536_ stability.

Many PAIs share features with lysogenic bacteriophage genomes and it has been discussed that PAIs may have evolved from former lysogenic bacteriophages by gradually losing some of their phage characteristics. Thus IHF-dependent regulation of integrase gene expression, together with the role of both, integrase and IHF, as members of the recombinogenic complex involved in site-specific recombination may be a relic of the “former life style” of PAI I_536_ as a selfish mobile genetic element. Clearly, the seven PAIs in *E. coli* strain 536 evolved from different ancestral phages. According to the amino acid sequence, the PAI I_536_-encoded integrase is more closely related to the P4 integrase than the other PAI-encoded integrases in UPEC strain 536 (Figure S4), which may explain the specific role that IHF plays for the stability of PAI I_536_.

## Methods

### Bacterial strains and plasmids used in this study

The bacterial strains used in this study are listed in Table S2. The plasmids used in this study are listed in Table S3.

### Construction of strains and plasmids

#### Construction of fis, ihfA, ihfB and ihfA/B mutants

The *fis*, *ihfA*, *ihfB* and *ihfA/B* deletion mutants of our previously described reporter strains of *E. coli* strain 536 enabling the analysis of PAI I_536_-VI_536_ stability and of control strain 536 TR tagged with the P*dps* module^37^ were constructed using the Red/ET recombineering method^61^. For the replacement of *fis* and the *ihfA* by *a* zeocin resistance cassette, the template pKD8 and primer pairs MC38/MC39 and MC46/MC47 were used, respectively. For the replacement of *ihfB* with a gentamicin resistance cassette, the template pKD11 and primer pair MC48/MC49 were used. Double mutants for *ihfA/B* were constructed by first replacing *ihfA* with a zeocine resistance cassette and afterwards *ihfB* with a gentamicin cassette as described above. The replacement was afterwards controlled by PCR using primer pairs MC60/MC61, MC68/MC69 and MC70/MC71 as listed in Table S1. The obtained PCR products were additionally Sanger sequenced.

#### Construction of *E. coli* 536 *int*I-*yfp*

For the construction of a chromosomal *int-yfp* operon the *yfp-ble* segment was PCR amplified with Q5 High-Fidelity DNA Polymerase using primers MC138 and MC139 and pBAD24*yfp*-*ble* as template. The chromosomal fusion to *int*I was afterwards done by RedE/T recombineering^61^. The 5′and 3′ junctions were afterwards verified with PCR using the primer pairs MC140/MBP206 and 1720/MC14, respectively. The nucleotide sequence of the obtained PCR products was additionally determined by Sanger sequencing.

#### Construction of pWKS30*ihfB*

The plasmid pWKS30*ihfB* was constructed by amplifying *ihfB* with Q5 High-Fidelity DNA polymerase using primer pairs MC123/MC71 and chromosomal DNA of *E. coli* strain 536 as template. The PCR product was then cloned into *Eco*RV-HF digested low copy plasmid pWKS30^62^. Verification of pWKS30*ihfB* was done by PCR using primer pair MC111/MC112 and Sanger sequencing.

#### Construction of pBAD24*yfp*-*ble*

Construction of the intermediate pBAD24*yfp*. The *yfp* fragment was PCR amplified with Q5 High-Fidelity DNA polymerase using the primer pair 69_s/385. The PCR product was then digested with *Nco*I/*Xba*I and the 758-bp fragment cloned into the previously *Nco*I/*Xba*I digested vector pBAD24. The resulting plasmid was isolated and verified by analytical digestion, PCR using the control primers 733 and 734 to amplify the whole insert, and by Sanger sequencing (Table S1). The plasmid was named pBAD24*yfp*. The plasmid pBAD24*yfp*-*ble* was constructed by subcloning the *ble* cassette from the *Xba*I digested pKD8. The 748-bp *ble* fragment was subcloned into the *Xba*I digested pBAD24*yfp*. The plasmid was isolated and verified by analytical digestion, PCR using the control primers 733 and 734 to amplify the whole insert, and by Sanger sequencing (Table S1).

#### Construction of pKD8 and pKD11

The plasmid pKD8 was constructed by subcloning the *ble* cassette from the *Eco*RV-HF/*Pvu*II-HF digested plasmid pEM7/Zeo into the *Pvu*II-HF/*Nae*I digested pKD4 backbone. The plasmid was transformed into *E. coli* strain SY327*λpir*, isolated and verified by analytical digestion, PCR using the control primers 1074 and 1092 to amplify the whole insertion, and by Sanger sequencing (Table S1).

The plasmid pKD11 was constructed by subcloning the gentamicin cassette from the *Nco*I-HF/*Sac*II digested plasmid pBBR-1MCS-5; a fragment of 828 bp was processed with Mung Bean nuclease and subsequently cloned into the *Pvu*II-HF/*Nae*I digested pKD4 backbone. The plasmid was transformed into *E. coli* strain SY327*λpir*, isolated and verified by analytical digestion, PCR using control primers 1074 and 1092 to amplify the whole insertion, and by Sanger sequencing (Table S1).

#### Construction of pMC1 and pMC2

The plasmid pMC1 was constructed by amplifying the *int*I integrase gene of PAI I_536_ (including the IHF binding site) with Q5 High-Fidelity DNA polymerase using primers MC115 and MC14 and chromosomal DNA of *E. coli* strain 536 as template. The plasmid pMC2 was constructed by amplifying *int*I (excluding the IHF binding site) with Q5 High-Fidelity DNA polymerase using primer pair MC124/MC14 and chromosomal DNA of *E. coli* strain 536 as template. The PCR products were cloned into *Eco*RV-HF digested low copy plasmid pWKS30. Verification of pMC1 and pMC2 was done by PCR using the primer pair MC111/MC112 and by Sanger sequencing.

#### Construction of pMC3

The plasmid pMC3 was constructed by amplifying the *int*I*::yfp-cat* construct with Q5 High-Fidelity DNA polymerase using primer pair MC11/MC14 and chromosomal DNA of *E. coli* strain 536 PAI I *int*I*::yfp-cat*^37^ as template. The PCR product was cloned into *Eco*RV-HF digested low copy plasmid pWKS30. Verification of pMC3 was done by PCR using the primer pairs MC111/MBP206 and MB5/MC112 to control 5´- and 3´-junctions of insert and plasmid, respectively, as well as by Sanger sequencing.

#### Construction of pMC5 and pMC7

The plasmid pMC5 was constructed by amplifying the *int-yfp* operon with Q5 High-Fidelity DNA polymerase using primers MC125 and MC14 and chromosomal DNA of *E. coli* strain 536 *int*I*-yfp* as template. The PCR product was cloned into *Eco*RV-HF digested low copy plasmid pWKS30. The plasmid pMC7 was constructed by amplifying the *int*I*-yfp* operon with Q5 High-Fidelity DNA Polymerase using primer pair MC124/MC14 and chromosomal DNA of *E. coli* strain 536 *int*I*-yfp* as template. The PCR product was cloned into *Eco*RV-HF digested low copy plasmid pWKS30. Verification of both plasmids was done with the primer pairs MC111/MBP206 and 1720/MC112 to control 5′- and 3′-junctions of insert and plasmid, respectively, as well as by Sanger sequencing.

### Construction of pUC19P*int* and pUC19P*int* ∆IHFbs

The plasmid pUC19P*int*I was constructed by amplifying the *int*I promoter region with Q5 High-Fidelity DNA polymerase using primers MC125 and MC14 and chromosomal DNA of *E. coli* strain 536 *int*I::*yfp-cat*as template^37^. The PCR product was cloned into the *Sma*I digested high-copy vector pUC19. The plasmid pUC19P*int*I*∆*IHFbs was constructed by amplifying the *int*I promoter region with Q5 High-Fidelity DNA polymerase using primer pair MC124/MC14 and chromosomal DNA of *E. coli* strain 536 as template^37^. The PCR product was cloned into the *Sma*I digested high-copy vector pUC19. Verification of both plasmids was done with primer pairs 385/MBP206 and 384/MBP5 to control 5′- and 3′- junctions of insert and plasmid, and by Sanger sequencing.

#### Computational mapping of IHF binding sites

The computational mapping of IHF binding sites was performed as previously described^63^. The IHF consensus binding sequence was created based on the 124 sequences available in the TF binding sites experimental dataset on RegulonDB^64^. The 13-nt long consensus sequence was generated with MEME^65^ and then submitted to FIMO^66^ for scanning the occurrence of motifs with p  <  0.0001 in *E. coli* strain 536 genome. The list of IHF binding site candidates was further examined for motifs found in the vicinity of *int*I-VI.

#### Electric mobility shift assays (EMSA)

scIHF was purified from plasmid pETscIHF2 as described previously^40^. The protein concentration was determined using the micro BCA protein assay kit (Thermo Fisher Scientific) and the quality of the protein preparation was controlled using SDS-PAGE (Fig. S2). A digested plasmid offers DNA fragments that do have binding sites and other fragments that are not expected to have binding sites in stoichiometric amounts, i.e. this experimental setup has the advantage of internal positive, as well as negative controls. The binding reaction was performed at 37 °C for 5 min by premixing a constant amount of digested pUC19P*int*I and pUC19P*int*I ∆IHFbs vectors (250 ng) with the indicated amount of purified protein (0.5 to 0.2 µg) in 20 µl final volume of STE buffer (100 mM NaCl, 10 mM Tris-Cl, pH8, 1 mM EDTA). Afterwards, the binding reactions were loaded on a 1.5% TAE agarose gel and run at 130 V for 3 h. The DNA was then visualized by EtBr staining.

Additionally, PCR products of the *int*I promoter region were generated with Q5 High-Fidelity DNA polymerase using primers MC125/MBP206 and MC124/MBP206, and the plasmids pUC19P*int*I and pUC19P*int*I ∆IHFbs as templates, respectively. EMSAs were carried out using the DIG Gel Shift Kit, 2^nd^ Generation (Sigma-Aldrich, Taufkirchen, Germany) according to the manufacturer’s instructions. 25 ng of DIG-labeled probes were mixed with decreasing concentrations of purified scIHF (80 to 20 µg), and the reaction mixture was incubated at room temperature for 30 min. Protein-DNA complexes were then applied to an 8% (w/v) polyacrylamide gel, run for 2 h at 100 V in 0.5x TBE buffer using the Owl Dual-Gel Vertical Electrophoresis Systems (Thermo Fisher Scientific) and then electrically transferred to a nylon membrane. The DIG-label were then detected by chemiluminescence using the ChemiDoc MP System (BioRad, Munich,Germany).

#### Media

Lysogeny broth (LB; containing 10 g/L tryptone, 5 g/L yeast extract, 5 g/L NaCl) was used as standard growth medium. For the solid medium preparation, agar was added to a final concentration of 1.5% (w/v). When required, the medium was supplemented with the appropriate concentration of the selected antibiotic. Phosphate buffered saline (PBS; containing 8 g/L NaCl, 0.2 g/L KCl, 1.42 g/L Na_2_HPO_4_, 0.27 g/L KH_2_PO_4_, pH 7.4) was used for the washing step and for resuspending bacterial cells prior flow cytometric measurements.

#### Flow cytometry and cell sorting

Flow cytometric measurements were done using a Gallios flow cytometer (Beckman Coulter Life Sciences, Krefeld, Germany) equipped with a blue air-cooled argon laser (488 nm) and fluorescence filter sets for fluorescence FL1 (BP 515–545 nm) and FL3 (LP > 650 nm). For sorting of bacterial cells we used the BD FACSAria III model cell sorter (Becton-Dickinson Biosciences, Heidelberg, Germany). The instrument settings have been made utilizing the BD FACSDiva software. The sample preparation and the programs of the instruments have been described previously^37^. Briefly, the samples for the measurement of the stability of the islands in *E. coli* 536 wild type and different isogenic variants were prepared from independent overnight cultures grown under standard laboratory conditions and inactivated with an equal volume of 70% (v/v) isopropanol. For the measurements of the integrase promoter activity in its native position or when cloned into the different newly created vectors bacterial samples transitioning from logarithmic to stationary phase were used and prepared for the measurements as described above and elsewhere^37^.

#### Standard fluorescence signal detection and OD_595_ measurements

The samples were grown in LB at 37 °C under shaking standard laboratory conditions until the transition from logarithmic to stationary growth phase, and then prepared for the measurement. Prior each measurement 1 mL from every independent culture was washed once with PBS and re-suspended into an equal volume of PBS. The average fluorescence intensity as well as the optical density was measured with the TECAN infinite M200 plate reader (TECAN, Männedorf, Switzerland). Optical density was measured at 595 nm and the fluorescence signal measured using an excitation wavelength of 514 nm and detecting the emission signal at 550 nm with a detector gain of 100. Optical density signal was corrected for blank and fluorescence signal was corrected for background fluorescence signal (*E. coli* K-12 MG1655 for all strains). Afterwards YFP expression (Fluo/OD_595_) was calculated by normalizing corrected fluorescence signal (Fluo) to corrected optical density (OD_595_).

## Supplementary information

Supplementary Information.
