# Supplementary material for: IHF stabilizes pathogenicity island I of uropathogenic Escherichia coli strain 536 by attenuating integrase I promoter activity
Source: Sci Rep. 2020 Jun 10;10:9397. doi: 10.1038/s41598-020-66215-2 (PMC7286903; doi:10.1038/s41598-020-66215-2)
Supplement: Supplementary file 1 — Supplementary Information. [file 41598_2020_66215_MOESM1_ESM.pdf]

Supplementary information

IHF stabilizes pathogenicity island I of uropathogenic *Escherichia coli* strain 536 by attenuating integrase I promoter activity

Marco Chittò[1,2], Michael Berger[1], Petya Berger[1], Luisa Klotz[3], Peter Dröge[4], Ulrich Dobrindt[1,2]

[1]Institute of Hygiene, University of Münster, Münster, Germany
[2]Interdisciplinary Center for Clinical Research, University of Münster, 48149 Münster, Germany
[3]Department of Neurology with Institute of Translational Neurology, University Hospital Münster, 48149 Münster, Germany
[4]School of Biological Sciences, Nanyang Technological University, 637551, Singapore

Supplementary material and methods

General information

All the primers used in this study were purchased from Sigma-Aldrich (Taufkirchen, Germany) and are listed in Table S1. All bacterial strains used in this study are listed in Table S2. All plasmids used this study are listed in Table S3. All the restriction enzymes and the T4 DNA ligase were purchased from New England Biolabs (Frankfurt, Germany). The relevant parts of all constructs were Sanger sequenced after analytical PCR for the chromosomal constructs and restriction digestion for the plasmid constructs.

Supplementary Table S1. Primers used in this study

Primer designation	Sequence (5'→3')
MBP5	GGATGAATGGCAGAAATTCG
MBP206	GCTGAACTTGTGGCCGTTTA
MC11	TGATCTTCCGCCAACTAACC
MC14	GTACAACGTTATTCTCAATA
MC38	GGCATACTTCGAAAATTTTGCGTAAACAGAAATAAAGAGCTGACAGAACTGTGTAGGCTGGAGCTGCTTC
MC39	CTTCCCCATGCCGAGTAGCGCCTTTTTAATCAAGCATTTAGCTAACCTGAACATATGAATATCCTCCTTA
MC46	TGTAGAGGCATTAAAAGAGCGATTCCAGGCATCATTGAGGGATTGAACCTGTGTAGGCTGGAGCTGCTTC
MC47	CGACAGTGAAAAGAAAAAAGGCCGCAGAGCGGCCTTTTTAGTTAGATCAGACATATGAATATCCTCCTTA
MC48	AATGCAGCAACAGCAGCCGCTTAATTTGCCTTTAAGGAACCGGAGGAATCGTGTAGGCTGGAGCTGCTTC
MC49	GAAAAAAGCACCCGACAGGTGCTTTTCTCGCGTTCAAGTTTGAGTAAAAACCATATGAATATCCTCCTTA
MC60	ATCTGGACACTGGGGAGTTG
MC61	TTTCCAGGCTTCGATCAGAC
MC62	ATGAAAGGGAAGAGCCATGA
MC63	CGCGTCTTTTCTGGCTAATTT
MC68	ACCAGTCACCTGGCAAAATC
MC69	TGGCGTAAATCAGGTAGTTGG
MC70	TGCAAACTTCTCCAACAACG
MC71	TTTGTTGAACGGTTGTCTGG
MC107	CGTGTATTAGGCGGAAAAAAC
MC108	GTCCGGTGCAGATAAAATGC
MC111	CTCTTCGCTATTACGCCAGC
MC112	CATTAATTGCGTTGCGCTCACT
MC115	GTAAACCATCCCTGCGAGAG
MC123	TAAAGGCGCAACCGTAGAAC
MC124	AGGGGTGTATTGGGGTATCA
MC125	TTTAACAACATCTTTGTTAT
MC138	CAATGGTACGCTGACTACATTGATTCACTCGCTTCGCAGACCTGAAACGAATGGTGTCTATCACTAAAGA
MC139	CCATCTGGCGGTTGAGGCAGGGTGGTTATTCATTATTCAGTGTCACTGAATGATTTAATCTGTATCAGGC
MC140	GGCACGGTTTCAGACACACT
69_s	CATGCCATGGTGTCTATCACTAAAGATCAA
384	ACGACGTTGTAAAACGACGG
385	AGGAAACAGCTATGACCATG
733	ATCACGGCAGAAAAGTCCAC
734	CTTCTCTCATCCGCCAAAAC
1074	CATATGAATATCCTCCTTAGTTCC
1092	GTGTAGGCTGGAGCTGCTTC
1720	GACTTCGTGGAGGACGACTT

Supplementary Table S2. List of bacterial strains used in this study

***E. coli* strains**	**Description**	**Reference**
536	Uropathogenic wild type strain (O6:K15:H31)	Berger *et al*., 1982
536 PAI I_P*dps-yfp-cat*	P*dps-yfp-cat* inserted into PAI I_{536}	Chittò *et al*., 2019
536 PAI II_P*dps-yfp-cat*	P*dps-yfp-cat* inserted into PAI II_{536}	Chittò *et al*., 2019
536 PAI III_P*dps-yfp-cat*	P*dps-yfp-cat* inserted into PAI III_{536}	Chittò *et al*., 2019
536 PAI IV_P*dps-yfp-cat*	P*dps-yfp-cat* inserted into PAI IV_{536}	Chittò *et al*., 2019
536 PAI V_P*dps-yfp-cat*	P*dps-yfp-cat* inserted into PAI V_{536}	Chittò *et al*., 2019
536 PAI VI_P*dps-yfp-cat*	P*dps-yfp-cat* inserted into PAI VI_{536}	Chittò *et al*., 2019
536 TR_P*dps-yfp-cat*	P*dps-yfp-cat* inserted into TR	Chittò *et al*., 2019
536 PAI I_P*dps-yfp-cat fis::ble*	P*dps-yfp-cat* inserted into PAI I_{536} *fis* replaced with *ble*	This study
536 PAI II_P*dps-yfp-cat fis::ble*	P*dps-yfp-cat* inserted into PAI II_{536} *fis* replaced with *ble*	This study
536 PAI III_P*dps-yfp-cat fis::ble*	P*dps-yfp-cat* inserted into PAI III_{536} *fis* replaced with *ble*	This study
536 PAI IV_P*dps-yfp-cat fis::ble*	P*dps-yfp-cat* inserted into PAI IV_{536} *fis* replaced with *ble*	This study
536 PAI V_P*dps-yfp-cat fis::ble*	P*dps-yfp-cat* inserted into PAI V_{536} *fis* replaced with *ble*	This study
536 PAI VI_P*dps-yfp-cat fis::ble*	P*dps-yfp-cat* inserted into PAI VI_{536} *fis* replaced with *ble*	This study
536 TR_P*dps-yfp-cat fis::ble*	P*dps-yfp-cat* inserted into TR *fis* replaced with *ble*	This study
536 PAI I_P*dps-yfp-cat ihfA::ble*	P*dps-yfp-cat* inserted into PAI I_{536} *ihfA* replaced with *ble*	This study
536 PAI II_P*dps-yfp-cat ihfA::ble*	P*dps-yfp-cat* inserted into PAI II_{536} *ihfA* replaced with *ble*	This study
536 PAI III_P*dps-yfp-cat ihfA::ble*	P*dps-yfp-cat* inserted into PAI III_{536} *ihfA* replaced with *ble*	This study
536 PAI IV_P*dps-yfp-cat ihfA::ble*	P*dps-yfp-cat* inserted into PAI IV_{536} *ihfA* replaced with *ble*	This study
536 PAI V_P*dps-yfp-cat ihfA::ble*	P*dps-yfp-cat* inserted into PAI V_{536} *ihfA* replaced with *ble*	This study
536 PAI VI_P*dps-yfp-cat ihfA::ble*	P*dps-yfp-cat* inserted into PAI VI_{536} *ihfA* replaced with *ble*	This study
536 TR_P*dps-yfp-cat ihfA::ble*	P*dps-yfp-cat* inserted into TR *ihfA* replaced with *ble*	This study
536 PAI I_P*dps-yfp-cat ihfB::aac*(*3*)-*I*	P*dps-yfp-cat* inserted into PAI I_{536} *ihfB* replaced with *aac*(*3*)-*I*	This study
536 PAI II_P*dps-yfp-cat ihfB:: aac*(*3*)-*I*	P*dps-yfp-cat* inserted into PAI II_{536} *ihfB* replaced with *aac*(*3*)-*I*	This study
536 PAI III_P*dps-yfp-cat ihfB:: aac*(*3*)-*I*	P*dps-yfp-cat* inserted into PAI III_{536} *ihfB* replaced with *aac*(*3*)-*I*	This study
536 PAI IV_P*dps-yfp-cat ihfB:: aac*(*3*)-*I*	P*dps-yfp-cat* inserted into PAI IV_{536} *ihfB* replaced with *aac*(*3*)-*I*	This study
536 PAI V_P*dps-yfp-cat ihfB:: aac*(*3*)-*I*	P*dps-yfp-cat* inserted into PAI V_{536} *ihfB* replaced with *aac*(*3*)-*I*	This study
536 PAI VI_P*dps-yfp-cat ifhB:: aac*(*3*)-*I*	P*dps-yfp-cat* inserted into PAI VI_{536} *ihfB* replaced with *aac*(*3*)-*I*	This study
536 TR_P*dps-yfp-cat ihfB:: aac*(*3*)-*I*	P*dps-yfp-cat* inserted into TR *ihfB* replaced with *aac*(*3*)-*I*	This study
536 PAI I_P*dps-yfp-cat ihfA::ble ihfB:: aac*(*3*)-*I*	P*dps-yfp-cat* inserted into PAI I_{536} *ihfA* replaced with *ble*, *ihfB* replaced with *aac*(*3*)-*I*	This study
536 PAI II_P*dps-yfp-cat ihfA::ble ihfB:: aac*(*3*)-*I*	P*dps-yfp-cat* inserted into PAI II_{536} *ihfA* replaced with *ble*, *ihfB* replaced with *aac*(*3*)-*I*	This study
536 PAI III_P*dps-yfp-cat ihfA::ble ihfB:: aac*(*3*)-*I*	P*dps-yfp-cat* inserted into PAI III_{536} *ihfA* replaced with *ble*, *ihfB* replaced with *aac*(*3*)-*I*	This study

***E. coli* strains**	**Description**	**Reference**
536 PAI IV_P*dps-yfp-cat ihfA::ble ihfB:: aac*(*3*)-*I*	P*dps-yfp-cat* inserted into PAI IV_{536} *ihfA* replaced with *ble*, *ihfB* replaced with *aac*(*3*)-*I*	This study
536 PAI V_P*dps-yfp-cat ihfA::ble ihfB:: aac*(*3*)-*I*	P*dps-yfp-cat* inserted into PAI V_{536} *ihfA* replaced with *ble*, *ihfB* replaced with *aac*(*3*)-*I*	This study
536 PAI VI_P*dps-yfp-cat ihfA::ble ihfB:: aac*(*3*)-*I*	P*dps-yfp-cat* inserted into PAI VI_{536} *ihfA* replaced with *ble*, *ihfB* replaced with *aac*(*3*)-*I*	This study
536 TR_P*dps-yfp-cat ihfA::ble ihfB:: aac*(*3*)-*I*	P*dps-yfp-cat* inserted into PAI I_{536} *ihfA* replaced with *ble*, *ihfB* replaced with *aac*(*3*)-*I*	This study
536 PAI I_P*dps-yfp-cat intI::ble*	*int*I replaced with *ble*	Chittò *et al*., 2019
536 PAI I *int*I*::yfp-cat*	*int*I replaced with *yfp-cat*	Chittò *et al*., 2019
536 PAI I *int*I*::yfp-cat ihfB::ble*	*int*I replaced with *yfp-cat, ihfB* replaced with *ble*	This study
536 *int*I-*yfp-ble*(o)	*yfp* transcriptionally fused downstream of the coding region of *int*I	This study
DH5α	F^- ∆*lacU196* (Φ80 lacZ∆M15) *recA1 hsdR17*	Taylor *et al*., 1993
MG1655	*K-12* F^- λ^- *ilvG*$^-$ *rfb-50 rph-1*	Blattner *et al*., 1996
BL21(DE3)	F^- *ompT gal dcm lon* $hsdS_B(r_B^- m_B^-)$ [*malB*+]K-12(λ^S)	Studier and Moffat, 1986
SY327*λpir*	*thi-1 thr leu tonA lacY recA*	Miller and Mekalanos, 1988

Supplementary Table S3. List of plasmids used in this study

Plasmid	Description	Reference
pKD46	*bla repA*$_{101}$ (Ts) *araC araB*p $_{\gamma}$ β *exo*	Datsenko and Wanner., 2000
pBBR-1MCS-5	*aac(3)-I*, template plasmid	Kovach *et al*., 1995
pEM7/Zeo	*bla*,*ble*, template plasmid	Invitrogen
pKD4	cloning vector	Datsenko and Wanner., 2000
pKD8	*ble* cassette cloned from pEM7/Zeo into pKD4 with the replacement of *aph* cassette	This study
pKD11	*aac(3)-I* cassette cloned from pBBR-1MCS-5 into pKD4 with the replacement of *aph* cassette	This study
pBAD24	cloning vector	Guzman *et al*., 1995
pBAD24*yfp*	*yfp* template plasmid	Berger *et al*., 2016
pBAD24*yfp-ble*	*ble* cassette cloned from pKD8 into pBAD24*yfp*	This study
pWKS30	expression vector	Wang *et al*., 1991
pWKS30 *ihfB*	*ihfB* cloned from *E. coli* strain 536 into pWKS30	This study
pMC1	*int*I cloned from *E. coli* strain 536 into pWKS30	This study
pMC2	*int*I cloned from *E. coli* strain 536 excluding the IHF binding site into pWKS30	This study
pMC3	*int*I*::yfp-cat* cloned from *E. coli* strain 536 PAI I *int*I*::yfp-cat* into pWKS30	This study
pMC5	*int*I-*yfp* cloned from *E.coli* strain 536 *int*I-*yfp* into pWKS30	This study
pMC7	*int*I-*yfp* cloned from *E.coli* strain 536 *int*I-*yfp* excluding the IHF binding site into pWKS30	This study
pETscIHF2	expression vector	Bao *et al*., 2004
pUC19	*bla*, expression vector	New England BioLabs
pUC19P*int*I	P*int*I cloned from *E. coli* strain 536 PAI I *int*I*::yfp-cat* into pUC19	This study
pUC19P*int*I ΔIHFbs	P*int*I cloned from *E. coli* strain 536 PAI I *int*I*::yfp-cat* excluding the IHF binding into pUC19	This study

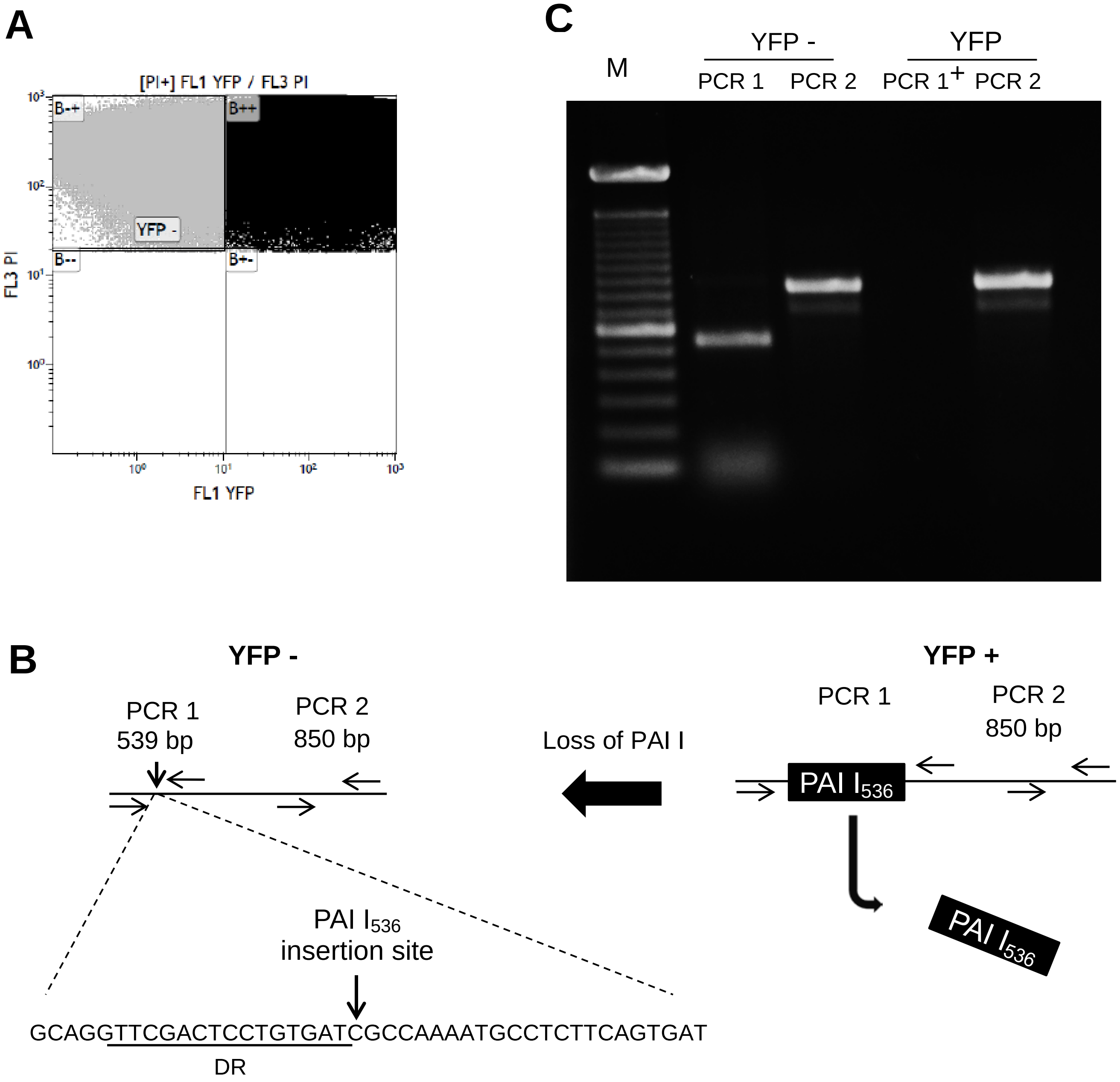

Supplementary Figure S1. Confirmation of chromosomal deletion of PAI I_{536} in YFP-negative cells. Shown UPEC 536 PAI I_P*dps-yfp-cat ihfA::ble ihfB::aac(3)-I*. **A)** Flow cytometric representation of the sample when combining the FL1 / FL3 channels; **B)** 10^5 YFP-positive and 10^5 YFP-negative bacterial cells were sorted (BD FACS Aria III, Becton-Dickinson Biosciences, Heidelberg, Germany) and analyzed with PCR. In YFP-negative cells (grey) a specific 593-bp PCR product can only be amplified with the primer pair MC115/MC108 when PAI I_{536} is deleted from the chromosome (PCR 1). This PCR product is absent in YFP-positive cells (black) where PAI I_{536} is inserted into the chromosome. As control, primers MC62 and MC63 allow amplification of an 850-bp PCR product (PCR 2) were used. Shown is also the sequence of the scar region with the chromosomal PAI I_{536} insertion site and the remaining repeat (DR). **C)** Detection of PCR products 1 and 2 in YFP-positive and -negative bacteria on a 1% agarose gel. Marker,100-bp ladder.

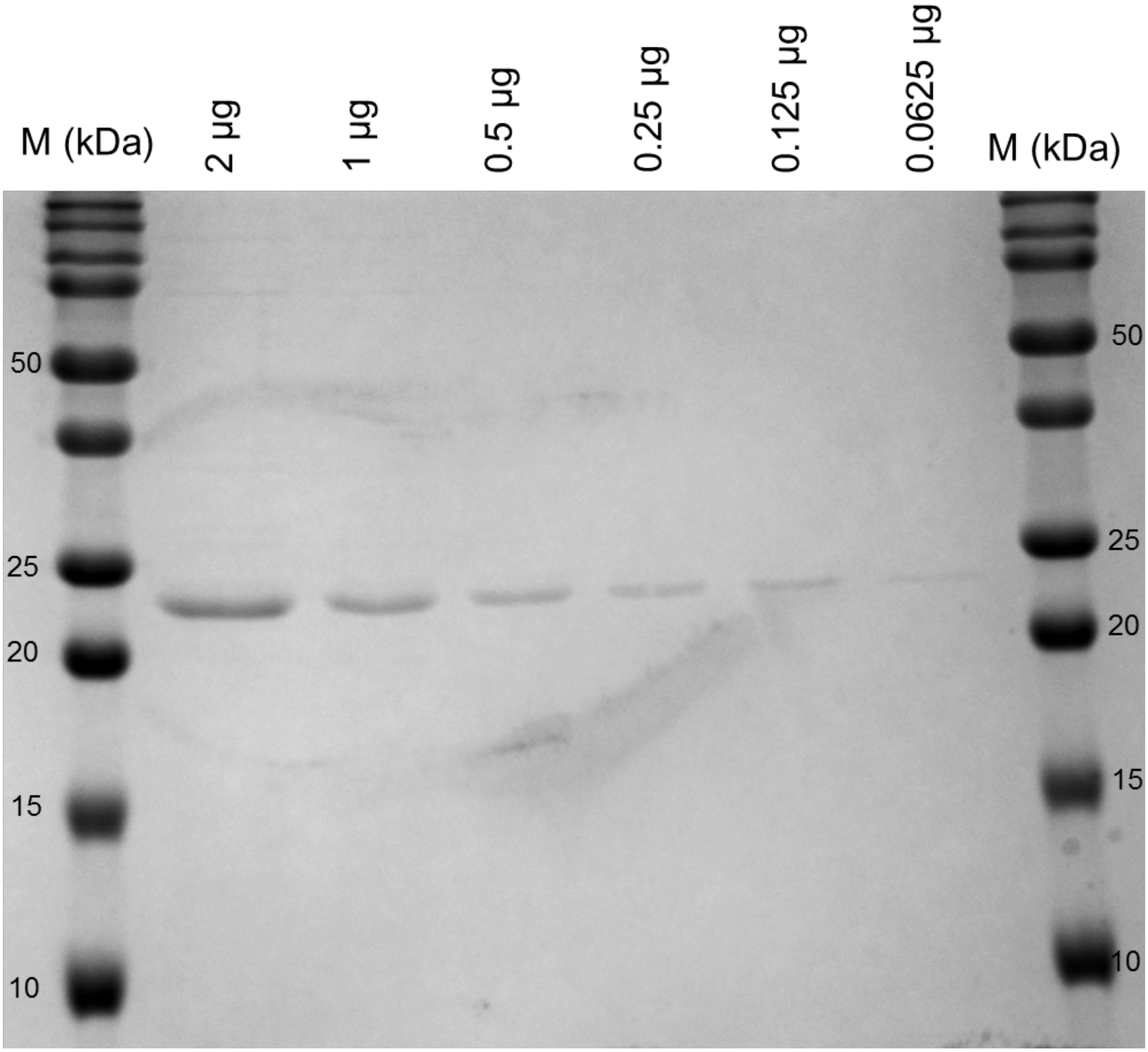

Supplementary Figure S2. Control of purified scIHF2 by sodium dodecyl sulfate polyacrylamide gel electrophoresis (SDS PAGE). scIHF2 was purified as described elsewhere. The protein concentration was determined using the BIO-RAD protein assay kit. Purified scIHF2 protein was analyzed in a 15% SDS-polyacrylamide gel together with molecular weight markers (Precision Plus Protein Dual Color Standards) (M) at the left and right side of the Coomassie-stained gel.

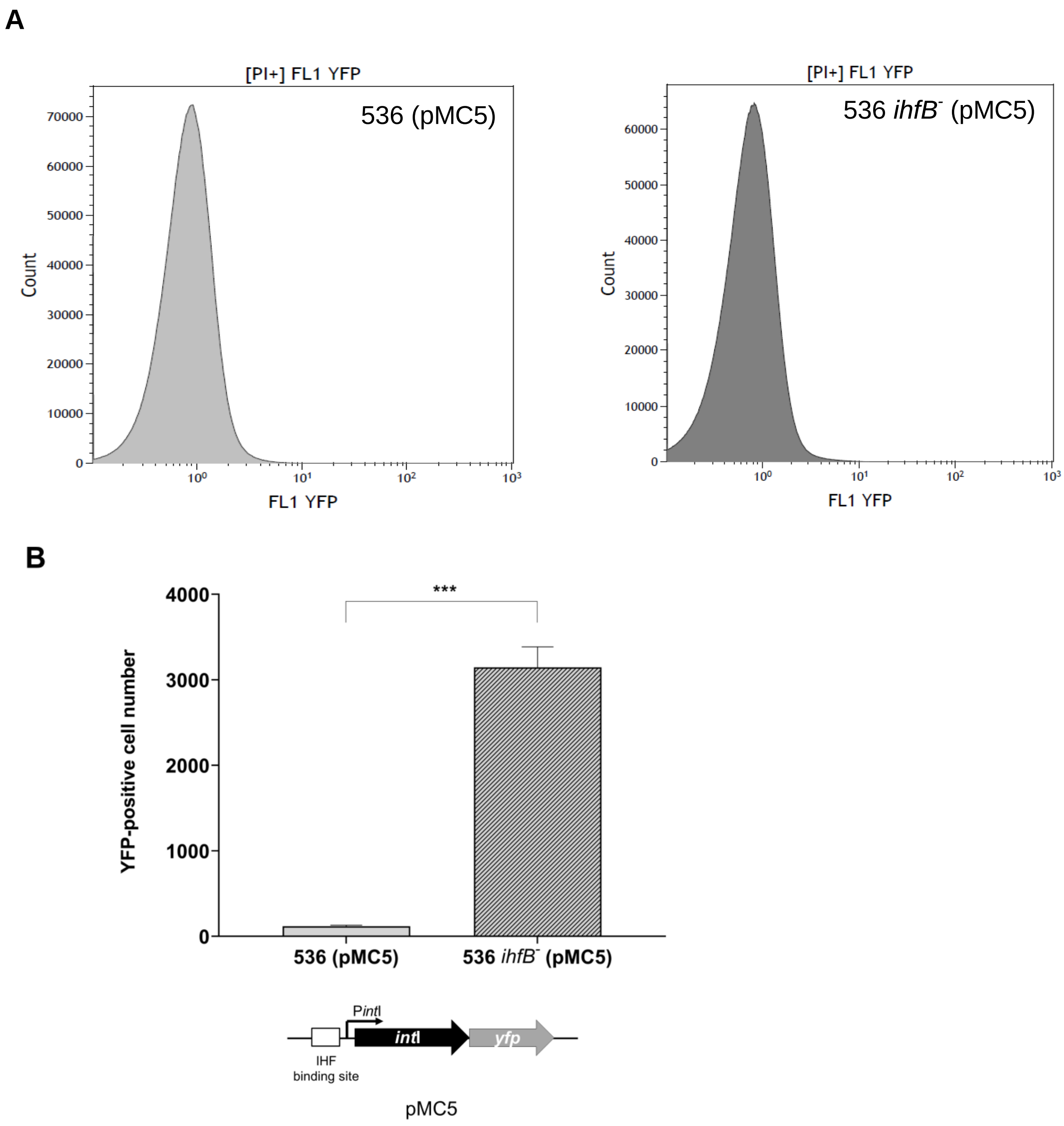

Supplementary Figure S3. Features of the fluorescence signals of *E. coli* strains 536 (pMC5) and 536 *ihfB*⁻ (pMC5). A) Shown is the YFP fluorescence signal distribution within the bacterial populations of *E. coli* strains 536 (pMC5) (light grey) and 536 *ihfB*⁻ (pMC5) (dark grey). 10^7 bacterial cells per sample were analyzed. **B)** Shown is the number of YFP-positive cells within the bacterial populations of strains 536 (pMC5) (light grey) and 536 *ihfB*⁻ (pMC5) (dark grey). The *ihfB* mutant population contains a significantly higher number of cells with a strong fluorescence signal than the wild type population. The columns represent the average of three biological replicates, in which 10^7 cells per sample were analyzed (* P<0.05;** P<0.01;*** P<0.001).

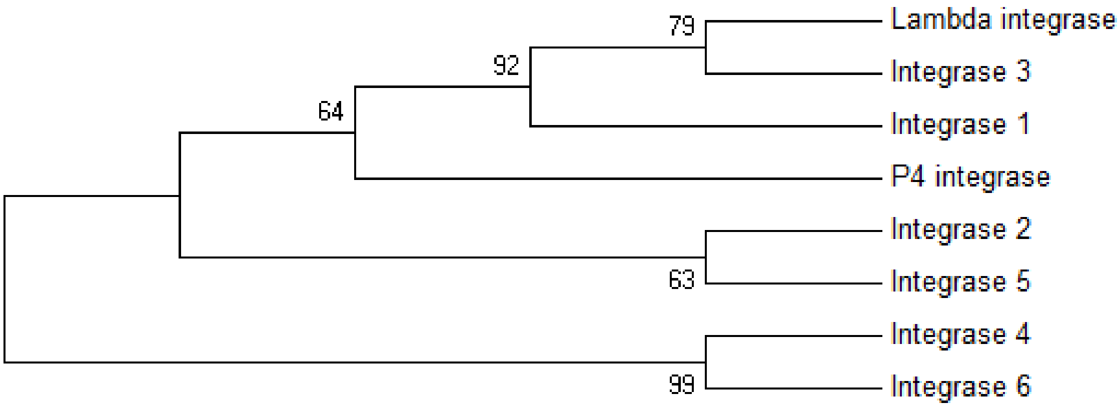

Supplementary Figure S4. Relatedness of PAI-encoded integrases of *E. coli* strain 536. The relatedness of PAI I_{536}-VI_{536}-encoded integrases of *E. coli* strain 536 was calculated (Maximum Likelihood method based on the JTT matrix-based model[1] in MEGA7[2]. The integrases of bacteriophage λ (lambda) and P4 were included as references. The bootstrap consensus tree was inferred from 1000 replicates. Branches corresponding to partitions reproduced in less than 50% bootstrap replicates are collapsed. The percentage of replicate trees in which the associated integrases clustered together in the bootstrap test (1000 replicates) are shown next to the branches[3]. Initial tree(s) for the heuristic search were obtained automatically by applying Neighbor-Join and BioNJ algorithms to a matrix of pairwise distances estimated using a JTT model, and then selecting the topology with superior log likelihood value. The analysis involved eight amino acid sequences. All positions containing gaps and missing data were eliminated. There were a total of 351 positions in the final dataset.

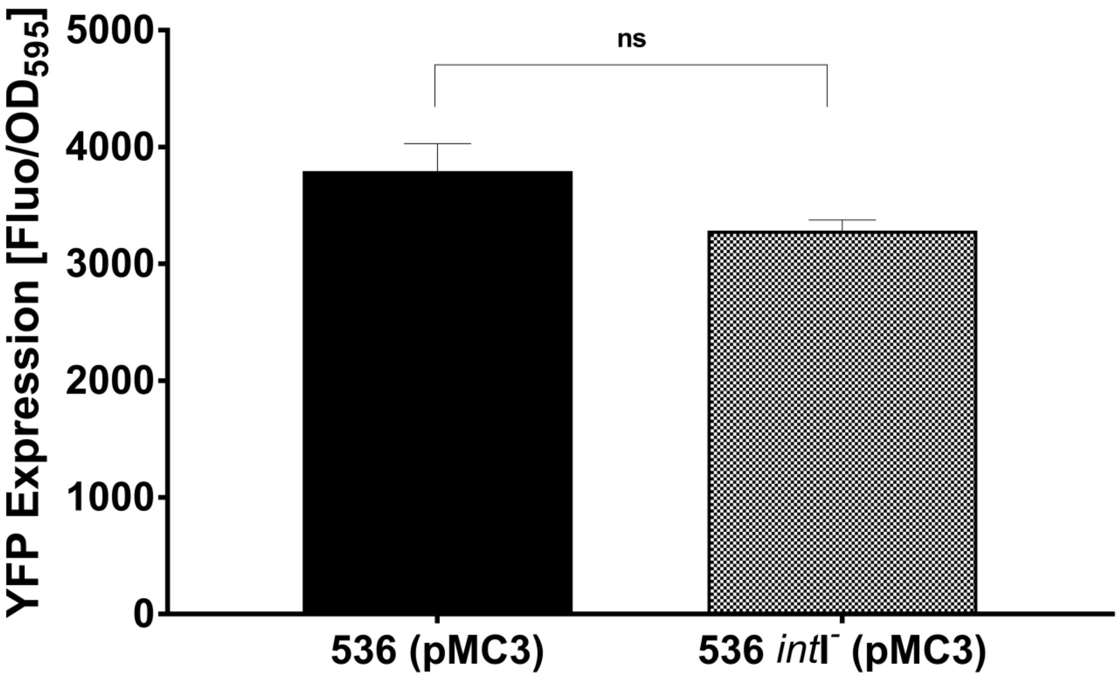

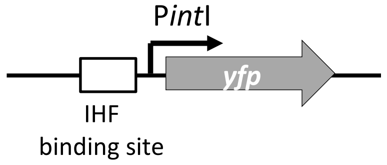

pMC3

Supplementary Figure S5. Comparison of the fluorescence signals of *E. coli* strain 536 (pMC3) and *E. coli* strain 536 *int*$^{-}$ (pMC3) Shown is the YFP fluorescence signal of *E. coli* strain 536 (pMC3) and *E. coli* strain 536 *intI*$^{-}$ (pMC3) normalized to the cell density (OD_{595}). The overall fluorescence signal of *E. coli* strain 536 (pMC3) and *E. coli* strain 536 *intI*$^{-}$ (pMC3) is very strong and the measured values are comparable. The columns represent the average of three biological replicates (* P<0.05;** P<0.01;*** P<0.001).

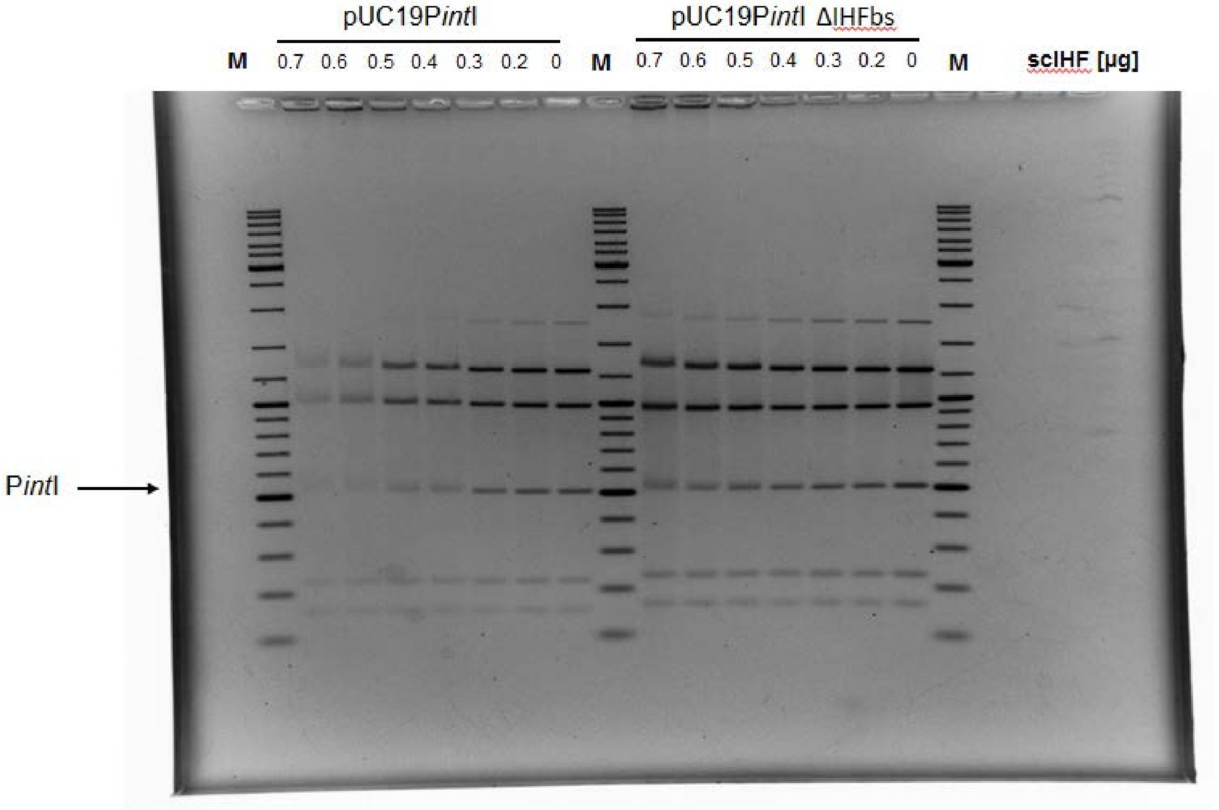

Supplementary Figure S6. Analysis of scIHF binding to the *int*I upstream region by electrophoretic mobility shift assay (EMSA). Single-chain IHF bound specifically to the promoter fragment (P*int*I) of pUC19P*int*I or pUC19P*int*IΔIHFbs only in the presence of the IHF binding site (left), but not when the binding site was absent (right). M, DNA size marker. The position of the P*intI* containing DNA fragment is indicated.

Supplementary literature

1. Jones, D. T., Taylor, W. R. & Thornton, J. M. The rapid generation of mutation data matrices. *Comput Appl Biosci.* **8**, 275–282 (1992).

2. Kumar, S., Stecher, G. & Tamura, K. MEGA7: Molecular Evolutionary Genetics Analysis Version 7.0 for Bigger Datasets. *Mol. Biol. Evol.* **33**, 1870–1874 (2016).

3. Felsenstein, J. Confidence Limits on Phylogenies: an Approach Using the Bootstrap. *Evolution (N. Y).* **39**, 783–791 (1985).
